# Management staff’s perspectives on intervention strategies for workplace violence prevention in a tertiary health facility in Nigeria: a qualitative study

**DOI:** 10.3389/fpubh.2023.1210571

**Published:** 2023-08-15

**Authors:** Adaoha Pearl Agu, Benedict Ndubueze Azuogu, Alfred F. Una, Benard Ituma, Irene Ifeyinwa Eze, Francis I. Onwe, Onyinyechukwu Uzoamaka Oka, Dorathy O. Igwe-Okomiso, Urudinachi N. Agbo, Richard Ewah, Jesse C. Uneke

**Affiliations:** ^1^Community Medicine Department, Ebonyi State University Abakaliki, Abakaliki, Nigeria; ^2^Community Medicine Department, Alex Ekwueme Federal University Teaching Hospital Abakaliki, Abakaliki, Nigeria; ^3^African Institute for Health Policy and Health Systems Ebonyi State University Abakaliki, Abakaliki, Nigeria; ^4^Anaesthesia Department, Alex Ekwueme Federal University Teaching Hospital, Abakaliki, Nigeria

**Keywords:** workplace violence prevention, occupational health, tertiary health institution, qualitative study, Nigeria

## Abstract

**Introduction:**

Health workers have increasingly become victims of workplace violence. However, negligible action has been given to developing workplace violence (WPV) prevention programs in hospital settings in low-middle-income countries. An effective workplace violence prevention program is crucial for preventing violence and managing the consequences of incidents. This study assessed management staff perspectives on intervention strategies for workplace violence prevention in a tertiary health facility in Nigeria.

**Methods:**

A qualitative study design was employed to explore the intervention strategies for preventing and managing workplace violence at a tertiary health facility in southeast Nigeria. Six focus group discussions were conducted with thirty-eight management-level staff. The interview transcripts were manually coded according to six predefined constructs of workplace violence: creating interdisciplinary harmony and WPV experiences, causes, prevention, program/policy contents, and implementation strategies. A manual thematic analysis approach was adopted, and the results were presented as narratives.

**Results:**

The findings revealed recognition, welfare, administrative control, and security as vital strategies for the WPV prevention program. The participants agreed that unanimity among staff could be promoted through respect for all cadres of staff and for people’s perspectives (creating interdisciplinary harmony). Assaults and staff intimidation/victimization (experiences), attributed to unethical/poor health workers’ behaviour and ethnic discrimination (causes), were viewed as preventable by ensuring patients’/caregivers’ welfare through respectful and timely care and staff’s welfare through incentives/remunerations and discouraging intimidation (prevention strategies). Furthermore, the staff expressed that the WPV program should employ administrative controls, including instituting WPV policy/unit, codes of ethics, and standard operating procedures across all workplace facets (program/policy contents), which should be implemented through awareness creation, enforcement of sanctions, and provision of appropriate and adequate security presence in the hospital (policy implementation strategies).

**Conclusion:**

Respect, patient/staff welfare, administrative control, and security are strong mechanisms to prevent workplace violence in tertiary hospitals. Hospital management should institutionalize workplace violence prevention programs/policies and ensure compliance.

## Introduction

Workplace violence is incidents where staff are abused, threatened, or assaulted in the circumstances related to their work, including commuting to and from work involving an explicit or implicit challenge to their safety, well-being and health (1). Existing research suggests that healthcare workers are at a greater risk of exposure to workplace violence (2). Increasing reports of incidents among healthcare workers, the serious adverse effects on its victims, and the resulting socioeconomic cost have received considerable attention worldwide (3–5). Up to 38% of health workers are victims of physical violence at some point in their careers (6). Interestingly, most incidents can be prevented, and studies have reported developing and implementing interventions for control (7–9).

Guidelines have provided important information on strategies for preventing workplace violence in the healthcare sector (1). Furthermore, an effective workplace violence prevention program is key to preventing violence and managing the consequence of the incidents (10, 11). Earlier evidence describes initiatives to develop and implement programs for preventing and managing workplace violence in health care (4, 12). Among interventions to reduce bullying in healthcare organizations, participatory interventions yield the most promising outcomes, as shown in a scoping review on interventions to reduce workplace violence in a healthcare organization (8).

While the prevalence of workplace violence in healthcare settings has been documented in a few studies in Nigeria (13–15), there is a dearth of literature on intervention studies for the development of workplace violence prevention programs in a hospital setting in Nigeria. Thus, negligible action has been given to developing such important programs, particularly in low-resource settings (6). Such a program is imperative if the hazard of workplace violence is to be controlled.

Existing evidence suggests that healthcare workers are at great risk of exposure to workplace violence with serious adverse effects and socioeconomic costs on its victims (2). Regardless, little attention has been given to developing workplace violence prevention programs in hospital settings in low-middle-income countries (6). This is despite that an effective workplace violence prevention program is paramount for preventing and managing the consequences of workplace violence (9). Furthermore, there is limited literature on workplace violence prevention programs in a hospital setting in low-resource settings (6) including Nigeria. A scoping review showed that participatory interventions yielded promising outcomes in reducing workplace violence in a healthcare organization (8). Hence, the need to involve beneficiaries in developing policies and programs for workplace violence prevention. It is, therefore, noteworthy that this is the first study to the best of our knowledge that involved staff in designing intervention strategies for preventing workplace violence in tertiary health facilities in southeast Nigeria. This study aimed to assess management-level staff disposition on intervention strategies for workplace violence prevention in a tertiary health facility in Nigeria. The result would contribute to developing policies and programs to manage workplace violence issues in hospitals.

## Materials and methods

### Study design and setting

This study employed a qualitative design to explore management staff’s perspectives on intervention strategies for managing and preventing workplace violence in tertiary health institutions. The study area was Alex Ekwueme Federal University Teaching Hospital Abakaliki (AEFUTHA), a national tertiary health institution that resulted from a merger between the then Federal Medical Centre (FMC) and Ebonyi State University Teaching Hospital (EBSUTH), both in Abakaliki Ebonyi State in the year 2012. The hospital had about 4,000 staff at the time of the study, with 26 clinical and non-clinical departments. It is the second largest teaching hospital in the Southeastern part of Nigeria and is strategically located to serve three neighbouring states of Abia, Enugu, and Cross River.

### Study population, sampling method, and sample size

The population that participated in the study were the management staff of the hospital who gave informed consent to participate. Management-level staff absent on the day of the data collection (a day scheduled for a workshop) were excluded from the study. The participants were the management staff of the hospital, who were purposively selected as the group expected to be more knowledgeable about intervention strategies and could provide the required information.

To enable a representative sample size, the heads of the twenty-six departments in the hospital (clinical and non-clinical departments) were written to and requested to nominate two management staff each from all management staff in the unit; hence the purposive selection. Whereas some departments sent two nominees, some sent one, while others did not send any. A total of thirty-eight participants participated in the study, giving a response rate of 73%.

### Data collection processes

**Figure 1:**
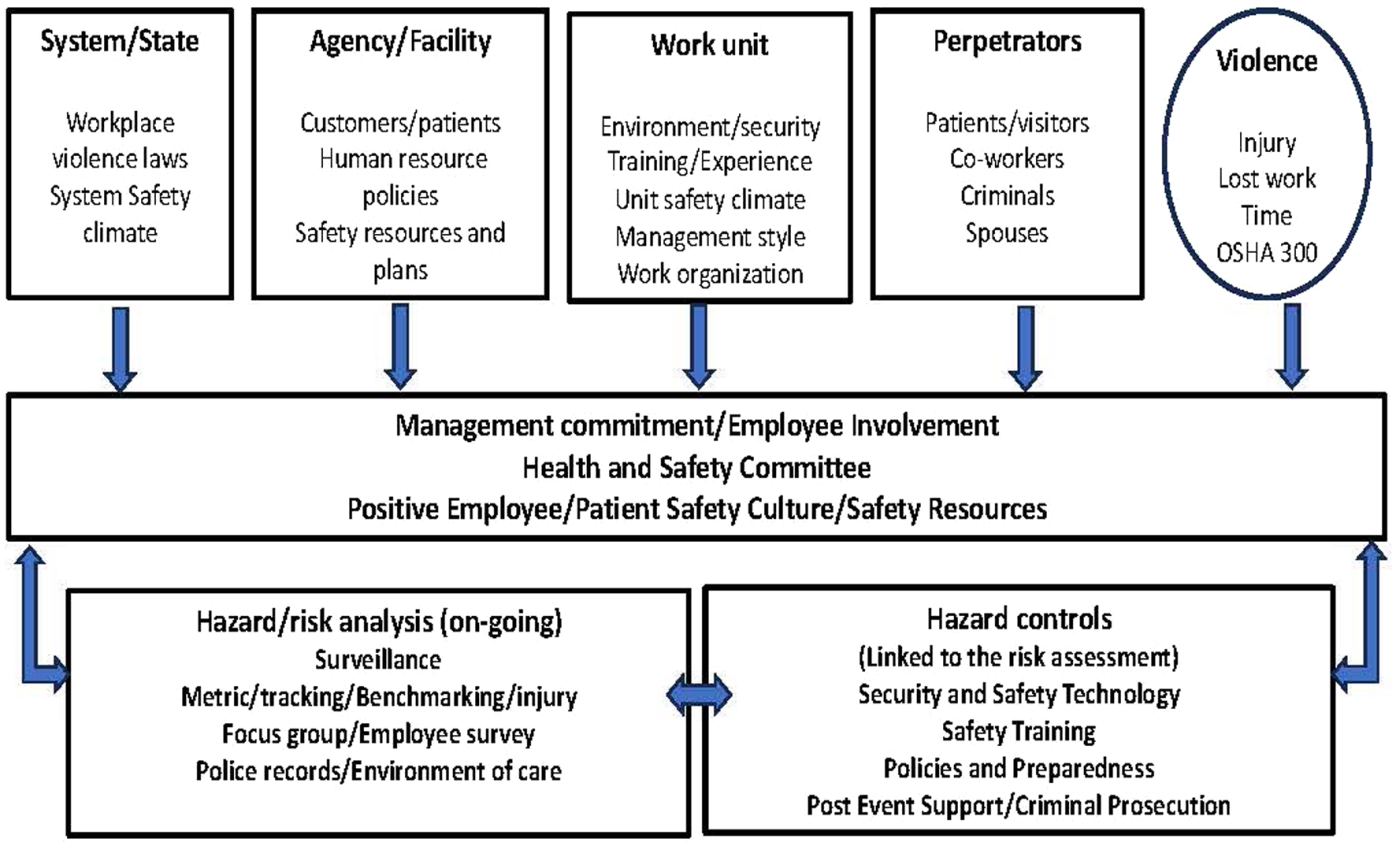
Framework for workplace violence prevention.

The data for the qualitative study was collected using focus group discussions (FGDs). The study instrument, the FGD question guide, was adapted from the “Framework for preventing workplace violence in the healthcare sector” (16) (Figure 1). The objective of the framework is to provide general guidance in addressing workplace violence in the health sector. Far from being in any way prescriptive, the framework is considered a basic reference tool for stimulating the autonomous development of similar instruments specifically targeted at and adapted to different cultures, situations and needs.

The question guide focused on specific areas related to the objectives of the study as adapted from the framework. Thus, the questions assessed participants’ perspectives on six topics/constructs related to intervention strategies for preventing workplace violence: (1) creating interdisciplinary harmony, (2) strategies to prevent workplace violence caused by patients, caregivers, visitors and staff, (3) components/contents of workplace violence prevention program, (4) implementation strategies by management to ensure staff compliance to policy on prevention of workplace violence, (5) experiences/examples of workplace violence, factors responsible and ways to address them (6) measures to be taken in cases of workplace violence. Prompts were used to explore further nuances about the topics to suit the local hospital context.

The data were collected during a one-day workshop organized for the management staff of AEFUTHA in March 2019 with the approval of the hospital management for this study. The FGD was conducted by the research team comprising the principal investigator – a female physician/researcher, and six other physicians and researchers from the Department of Community Medicine and the African Institute of Health Policy and Health Systems experienced in qualitative research.

Six focus group discussions were conducted. Participants were allocated into six groups stratified into clinical and non-clinical and medical and surgical departments, each group having six to eight participants. Members of the research team were assigned to the groups, two persons per group, a moderator, and a note taker. Many participants requested no audio recording to be free to make honest contributions, which was granted. Before the data collection, participants were informed of the purpose of the study and written informed consent was obtained by signing the consent form. Also, each group’s moderator recorded each participant’s demographic details and work-related characteristics (age, sex, marital status, duration in position, designation etc.). Each focus group lasted for 40–50 min.

To ensure rigour, the following was undertaken (a) the use of experienced personnel for the FGD, transcription, coding, and validation of findings where there were discrepancies, (b) stratification of the study population into homogenous groups to enable appropriate discussion and views, (c) invitation of management staff from all the departments to ensure adequate representation of views from all departments of the hospital; although all departments did not attend, there was adequate representation of the main sections- clinical (medical and surgical) and non-clinical, (d) provision of an information sheet about the study to all the participants and obtaining written informed consent for participation.

After the FGD, the management staff were trained on workplace violence: definition, types of workplace violence, expected professional behaviours in the hospital and consequences of workplace violence. The training workshop was aimed towards improving their knowledge of workplace violence; geared towards introducing the proposed workplace violence prevention program. The training was conducted using a PowerPoint presentation and lasted about 60 min.

### Data management and analysis

The transcribed note from the focus group discussions was collated and independently reviewed by two researchers – the lead (female) and the second (male), senior public health physicians for inconsistencies, and dissenting views were reconciled. A manual thematic analysis approach for qualitative data analysis was adopted, using deductive and inductive approaches. Initial deductive coding was based on the six pre-defined key themes (the six topic areas), and inductive coding was used to explore new/emerging themes that the topic areas did not cover. Two initial transcripts were read for familiarization, coded by the two senior researchers, and compared with the senior researcher to ensure coding consistency and comparability and facilitate collaborative thematic analyses. Themes and sub-themes were continually reviewed and refined to capture emerging codes. Thereafter, themes were merged into a coding framework structured to reflect the central research question of workplace preventive strategies, and these were: perceptions towards the creation of interdisciplinary harmony, strategies for workplace violence prevention, content of a workplace violence prevention program, policy/program implementation strategies, experiences of WPV and responsible and mitigation factors, and measures to be taken in cases of workplace violence. Quotes were captured to highlight thematic areas and increase our understanding of the context. The findings were then organized and reported as narratives.

### Ethical considerations

Ethical approval was obtained from the Research and Ethics Committee of Alex Ekwueme Federal University Teaching Hospital, Abakaliki (AEFUTHA), (Ref No: FETHA/REC/Vol2 2/2019) (REC approval No: 08/03/2019–10/03/2029). Permission was obtained from the top management of the hospital. Participants were informed of the purpose of the study and informed written consent was obtained by signing the consent form. Participation was voluntary. Confidentiality of responses was assured. The notes taken by the researchers had no personal identifiers like names attached to the responses and participants were assured that their responses would only be used for the study and to make recommendations. The participants had requested no audio recording which was granted. As such, the participants felt free to discuss among themselves, more so, as they are all the same status – management staff.

## Results

A total of 38 management staff participated in the study. The mean age was 39 ± 9.6 years. The majority were women (57.2%) and 71.4% were married. Fourteen departments (53.8%) in the hospital were represented: Medical Laboratory Science, Pharmacy, two out of the four departments in the Nursing Services Department (Nutrition and Dietetics, School of Nursing), six out of nine broad clinical departments (Internal Medicine, Haematology, Community Medicine, Dental Surgery, Pathology); four out of ten departments in Administration (Works, Cooperate Communication, Biomedical Engineering and General Administration). The findings from the six predefined constructs are presented below.

The results of four key question topics/construct focusing on the central research questions of workplace violence prevention – creating interdisciplinary harmony, strategies for preventing workplace violence, contents/components of WPV policy/program, and implementation of a workplace violence prevention program are shown in Table 1.

**Table 1:** Management staff’s perspectives on creating interdisciplinary harmony in workplace violence prevention programmes and implementation strategies.

Topic/constructs	Operational definitions	Themes and sub-themes
(1) Creation of interdisciplinary harmony	How to create inter-disciplinary harmony to prevent workplace violence caused by interdisciplinary rivalry	Promote respect and recognition for persons and views. Respect for people’s perspective Respect to other staff Respect to authorities Allow freedom of expression Encourage teamwork. Discourage staff intimidation.
(2) Strategies for prevention of workplace violence caused by caregivers, visitors, and staff	Strategies that management can put into place to prevent workplace violence caused by caregivers, visitors, and staff.	1. Institute packages to promote patient/caregiver’s welfare. Policy to attend timely to patients. Provision of a conducive environment for patients/caregivers. Provision of necessary work materials/equipment to caregivers. 2. Institute policy to promote staff welfare. Increase human resources. Provision of adequate remuneration (incentives) for the workforce. Policy to ensure diligent care to patients. 3. Institute policy to regulate visitors. i. Proper identification of visitors before permission to see a patient. ii. Proper screening of visitors before granting permission to see patients.
(3) Content of workplace prevention programme	What should be the components/content of workplace violence prevention programme	1. Institution of adequate and appropriate administrative control. i. Institution of code of ethics (anonymity, respect etc.). ii. Establishment of standard operation procedures (S.O.P) across all facets of the workplace. iii. Establishment of a monitoring and evaluation unit. Institution of sanctions for erring officers Adequate security personnel with requisite training 2. Establishment of workplace violence prevention program unit. i. Instituting occupational hazard committee ii. Provision of ideal office space, and functional equipment, supplies, and logistics (e.g., constant supply of water, light).
(4) Implementation strategies of proposed policy on workplace violence prevention	Implementation strategies that would ensure staff compliance to a proposed policy development on workplace violence prevention, and implementation.	Creating awareness about policy Enforcement of policy Ensuring uniformity in handling policy-related cases Stipulating penalties for lack of compliance Accessibility of managers to all staff. Provision of adequate security

## Creation of interdisciplinary harmony

Under the creation of interdisciplinary harmony, management staff identified four themes (with sub-themes) including (i) promoting respect and recognition for persons and views (respect for people’s perspective, respect to other staff, respect to authorities), (ii) allowing freedom of expression, (iii) encourage teamwork, and (iv) discourage staff intimidation.

**(i) Promote respect/recognition**: respect/recognition was a predominant deposition among respondents across topics exploring strategies for creating interdisciplinary harmony to prevent workplace violence. Respondents viewed that respect and recognition could be viewed in various forms like respect for people’s perspectives, professional respect, and respect for authorities, the respondents felt that disrespect for each other’s profession and across different cadres among health workers was a cause of workplace bullying. They viewed that respect and recognition are crucial in preventing workplace violence as it impacts other aspects of workers’ relationships. The underlying nuance was that doctors felt superior to others and often lacked professional respect for other professions and cadres.

*“Every profession should be given their respect and not a situation where one feels superior to the other*” (Non-clinical staff, Male).

Respondents unanimously agreed that orientation in professional respect among all the health worker cadre would create interdisciplinary harmony and prevent workplace violence.

**(ii) Allowing freedom of expression**: respondents linked respect to attitude towards colleagues as they viewed that recognition and respect impact staff’s attitudes towards freedom of expression. One respondent alluded to this notion thus:

“…*allow each person to present his opinion on issues and do not attach special importance to a particular profession when they speak*” (Non-clinical staff, Female).

**(iii) Encourage teamwork**: teamwork is a theme that appeared recurrently. Respondents agreed that working as a team has a synergistic effect, is more productive and is vital to preventing workplace violence. As one participant put it:

“…*we should learn to work together in this place; nobody is struggling for anything.”* (Clinical staff, Female).

**(iv) Discourage staff intimidation**: the theme of discouraging staff intimidation was recurrently expressed towards the creation of interdisciplinary harmony in the hospital. Respondents spoke to zero tolerance of workplace violence from one staff to another, which according to them, follows up on respect. The undertone was that some senior professionals intimidate junior ones. A respondent put it this way:

“*do not shout at people to get your way …People should not use their position to bully others. At times, we see and hear of threats in this place”* (Clinical staff, Male).

Respondents agreed that discouraging staff intimidation is vital in creating interdisciplinary harmony and admonished that staff should “avoid intimidation of others” to prevent workplace violence.

## Strategies for the prevention of workplace violence

The management staff suggested three strategies for the prevention of workplace violence caused by caregivers, visitors, and staff, presented under three themes and eight sub-themes. These include (i) institute policy and packages to promote patient/caregiver’s welfare with sub-themes – policy to appropriately (diligently, courteously and timely) attend to patients, and provide a conducive environment (including materials and equipment) for patients/caregivers; (ii) institute policy to promote staff welfare with sub-themes – increase human resources, provide adequate remuneration (incentives) for the workforce; and (iii) institute policy to regulate visitors with sub-themes – proper identification, and screening of visitors before granting permission to see patients.

**(i) Institute policies and packages to promote patient/caregiver welfare**: the institution of policy to ensure patients’ and caregivers’ welfare was a predominant theme that surfaced. The welfare of patients and their caregivers were considered vital to preventing workplace violence, as violence also emanates from patients and caregivers (not staff only).

**(a)** ***Policy to appropriately attend to patients***: respondents viewed that patients should be attended to promptly and diligently and courteously as this would promote cordial relationships and reduce misplaced aggression and other workplace violence in the hospital. They emphasized the need for a policy to ensure that patients are appropriately attended to and without delay. Talking about this issue, a participant stated. “*If the patient is treated on time, it will be good”* (Non-clinical staff, Female). In support of the opinion, another respondent said, “*A policy should be put in place to attend to patients as soon as they present in the hospital and not delay them*” (Non-clinical staff, Male).

In addition, respondents emphasized the need to be courteous and respectful to patients during interactions, and they should be made to feel important to reduce the level of aggression from them and their caregivers.

“*If the patient is treated as if he or she is something, they will not cause trouble.”* (Clinical staff, Male).

****(b)** *Provision of conducive environments for patients/caregivers***: respondents viewed the need for enabling environment for service provision. Some highlighted the need for providing a conducive environment for patients and caregivers as well as the necessary materials and equipment required to make patients and caregivers comfortable. They suggested that management should provide adequate reception room for visitors, functional equipment with a constant water supply, and light, to avoid transferred aggression. They expressed that those situations where patients’ treatment was delayed due to a power outage, as some had experienced, were unacceptable.

“…*somebody will come and spend hours in the hospital and not be comfortable*” (Clinical staff, Female).

**(ii) Institute policy to promote staff welfare**: the need for an increase in human resources in the hospital and the provision of adequate remuneration and incentives for the workforce were prominent strategies suggested to improve staff welfare specifically and to prevent workplace violence in general.

(**iii) Institute policy to regulate visitors**: identification and screening of visitors before permission is granted to see a patient were recurrent views of respondents. They opined that proper identification and screening of visitors upon entering the hospital should be adhered to, and there should be strict regulation and enforcement of visiting times. Restriction in the number of visitors/caregivers allowed at a time and provision of identification tags for visitors were opinions noted under this theme. They opined that as a last resort, security personnel can be viewed as first responders when violence occurs. A male non-clinical respondent stated, *“Map out clear times to see the patient that will be enforced.”*

However, a respondent although in agreement with the view for proper identification and screening of visitors, pointed out the need to be humane while conducting such exercises: “…*the visitors should be properly addressed and given the sense of belonging as this goes on.”* (Clinical staff, Female).

## Component/content of workplace prevention program

Two themes and seven sub-themes were suggested as the content of the workplace prevention program. They include (i) Instituting adequate and appropriate administrative control with the following sub-themes: (a) establishment of standard operation procedures (S.O.P) across all facets of the workplace, (b) institution of code of ethics, (c) establishment of a monitoring and evaluation unit, (d) enforcement of sanctions for erring officers, and (e) provision of adequate security personnel with requisite training; (ii) Establishment of a workplace violence prevention program unit which had as sub-theme (a) instituting occupational hazard committee and (ii) provision of ideal office space and functional equipment, supplies, and logistics.

**(i) Instituting adequate and appropriate administrative controls.**

The theme, instituting administrative control came up recurrently. Respondents outlined various administrative mechanisms to be set up, which, if in place, they believed, would make workplace violence difficult to thrive.

**(a)** ***Establishment of standard operation procedures (S.O.P)***: respondent emphasized the need for the establishment of S.O.P across all facets of the workplace. They noted that the hospital did not have an incident reporting or management system for workplace violence prevention program implementation. They highlighted the need to establish S.O.P across all facets of the workplace, including ensuring that people are treated as the come rather than allowing favouritism towards ethnic or clan interests in the management of cases.

*“…they should ensure the cases are treated first come, first served, and with the same hand* [measure], *not favouritism to those they know or come from their place.”* (Non-clinical staff, Female).

**(b)** ***Institution of code of ethics***: according to respondents, there is a need for the institution of code of ethics including maintaining anonymity, ensuring respect for all, equity, and justice. They emphasized that patients’ confidentiality and other ethical behaviour should be encouraged as positive supportive strategies. Regarding fairness and justice, they emphasized that all staff should have equal access to the WPV prevention unit, and discrimination in handling cases was decried. While advocating for uniformity in handling cases, the issue of ethnic or clan favoritisms was observed to be a common barrier to a healthy working environment; and there was a call to ensure there would be zero tolerance for unfair treatment in the policy.

**(c)** ***Establishment of a monitoring and evaluation unit***: respondents stated that a monitoring and evaluation unit is needed to monitor the activities of the workplace violence prevention program unit. The unit among other things will be expected to document cases of WPV and its management, analyze the data and make recommendations based on the findings to curb future occurrences. A respondent expressed:

*“We need a monitoring and evaluation unit that will cover the workplace. The unit will, among other things, oversee the frequency of the reports and how they are handled*” (Clinical staff, Female).

**(d)** ***Instituting and enforcement of sanction policy to erring staff***: the issue of sanctions for staff who default on the policy and the involvement of the hospital’s disciplinary committee was a strongly held view by many. A respondent stated:

*“If it is a case of staff against staff, they should refer them to the disciplinary committee. If they do not sanction such staff, others will continue doing it”* (Clinical staff, Female).

**(e)** ***Enhancing security presence in the hospital***: this theme revealed the respondents’ feelings about security presence in the hospital. Opinions differed on what was adequate. While some felt the need for an increased security presence to keep people on a short leash in check, some saw enhancing security as counterproductive, which can trigger aggression and fear. The latter group felt that proper training for those already recruited was adequate to prevent workplace violence.

**(ii) Establishment of workplace violence prevention program unit.**

The respondents expressed an immediate need for establishing a workplace violence prevention program unit (occupational hazard committee) for workplace violence prevention program implementation. They noted that the hospital did not have an incident or case reporting system and no designated team working on the WPV program or handling workplace violence issues. They emphasized that such units should be made to be functional with an organized committee manning the program.

The respondents pointed out the need for the provision of ideal office space and functional equipment, supplies, and logistics (e.g., constant supply of water, light).

## Implementation strategies of the proposed policy on workplace violence prevention

Under the implementation strategies of policy on workplace violence prevention, five themes were identified. These include (i) creating awareness about policy, (ii) enforcement of the policy, (iii) accessibility of managers to all staff, (iv) stipulating penalties for lack of compliance, and (v) provision of adequate security. Respondents unanimously agreed that the proposed policy on workplace violence prevention was overdue in the hospital, and when developed, the first task of the workplace violence program unit would be to create awareness about the policy and put measures in place to enforce this policy.

*“…the unit should put up write-ups/inscriptions at different parts of the hospital so that people will know what they are expected to know/do.”* (Clinical staff, Male).

Respondents emphasized that all staff should have easy access to this unit, and discrimination in handling cases was decried. While advocating for uniformity in handling cases, there was a call to ensure there would be zero tolerance for discrimination in the policy.

## Workplace violence experiences, factors responsible, and mitigating factors

The findings on management staff experiences of workplace violence, the responsible factors, and the mitigating strategies are presented in Table 2. Bullying, discrimination, and victimization were the most experienced workplace violence. The predominant recurring factors responsible for workplace violence were delays in patients’ treatment and ethnic discrimination. An adequate supply of working materials and avoiding intimidation were the predominantly suggested measures for mitigating workplace violence. Table 2 shows the experiences, responsible factors, and mitigating measures for workplace violence.

**Table 2:** Management staff’s experiences of workplace violence, responsible factors, and mitigating measures.

Experiences of workplace violence	Factors responsible for workplace violence	Mitigating measures for workplace violence
Assault	Delay in patients’ treatment due to lack of light.	An adequate supply of working materials
Discrimination	Unethical behaviour	Teamwork
Bullying	Ethnic discrimination	Avoid ethnic discrimination
Threats	Disclosing patient’s diagnosis	Maintaining confidentiality
Abuse	Lack of confidentiality	Avoid intimidation
Victimization		

### Measures to be taken in cases of workplace violence

Measures to be taken in cases of workplace violence are shown in Table 3. The result shows that hospital staff including the occupational hazard committee are expected to among other tasks, identify and manage cases and ensure security in different parts of the hospital. Patients are expected to report incidents that, if need be, can be referred to the occupational hazard committee. Caregivers should be appropriately identified and the number restricted. Finally, respondents opined that visitors should be identified, the number and visiting period regulated and rand adequate reception room provided for the visitors. The measures to be taken for workplace violence cases are shown in Table 3.

**Table 3:** Measures to be taken in cases of workplace violence.

Persons			
Staff	Patients	Caregivers	Visitors
Identify that there is an incident.	Incident report to the WVP system established.	Number of caregivers should be restricted.	Restriction of number of visitors
Occupational hazard committee established to look at how incident happened, preventive measures etc.	Patients’ restriction	Identifications made for caregivers	Visitor identification
Ensure security in different parts of the hospital.	Cases referred to the occupational hazard committee and roll out disciplinary measures.	Improvement in nursing care and other services	Visiting hours
When it is staff against staff, cases should be referred to disciplinary committee.			Providing adequate reception room for visitors

## Discussion

Our study highlights management staff’s disposition toward intervention strategies to create interdisciplinary harmony and prevent workplace violence in the hospital. Our study revealed that recognition/respect was a major theme in various forms. Respondents emphasized the need for the orientation of all cadres of health workers on professional respect to create interdisciplinary harmony. If we assume that professional disrespect, an issue among respondents, is an example of an at-risk behaviour to violence (which was the undercurrent from our findings), then training will likely have a positive effect as suggested in a scoping review on prevention and management of occupational violence and aggression in health care (4). A common intervention to reduce workplace violence is education with three key approaches: recognizing at-risk behaviours and triggers, communication and de-escalation, and evasive self-defence or breakaway training; evidence shows that the first two approaches have reduced violence incidence (4). Furthermore, the list of high-priority, feasible intervention strategies in the Occupational Safety and Health Administration (OSHA) guidelines for preventing workplace violence for healthcare and social service workers includes communicating risk using an organized process (12).

The health sector in Nigeria has been battling with issues of interdisciplinary disharmony, which was brought out to the fore in this study. Medical doctors are accused of looking down on other professions, which is usually denied, and they rather see themselves as victims of an unhealthy rivalry. So, one would wonder how much of a change in behaviour training would produce without other innovative strategies. Another aspect of disrespect mentioned in this study is from staff to patients. Poor staff attitudes, such as disrespect and abusive maternal care to patients (such as physical, verbal, and psychological abuse), have been reported globally but more so in low and middle-income countries (17, 18), and recommendation has been made for frequent in-service training on respectful maternal care among midwives (18). Respect between workers as a strategy *per se* did not make the OSHA list (12). However, it may be applied to the strategy of – “using registration to assist with identifying risky situations early” in a modified format by identifying professional disrespect as a risky situation. The lack of mention of professional respect or interdisciplinary harmony as a strategy buttresses the issue of local context being essential in recommending intervention strategies for workplace violence in any setting.

Workplace violence by current or former co-workers is widely believed to be the most prevalent form of workplace violence in the healthcare industry (3), and bullying behaviours, verbal abuse, and threats are common types (3). Staff prone to intimidating other staff should be flagged as being at risk of violence and severely warned. This was implied when respondents indicated that avoiding intimidation of other staff is a strategy to prevent workplace violence. Earlier evidence on risk factors facilitating the identification of patients at risk of committing workplace violence included a history of violence, being physically aggressive/threatening, and verbally hostile (4). While this is for patients, it may be applied to staff also since literature has shown that the most prevalent form of workplace violence is worker-to-worker. Defusing employees by peers on the OSHA list of intervention strategies may be useful if one notices an alert flag escalating/about to escalate among peers. Respondents who mentioned encountering threats from staff to staff during their work gave the impression that they were witnesses and neither the victims nor culprits. However, the contrary may have been the case as they were not pressed for details of possible personal experiences, which was outside the scope of this study. A scoping review indicated that workplace bullying, though not having a single meaning, may be defined as the systematic mistreatment of a subordinate or colleague by one or more individuals from the same group over a frequent (at least once a week) and long period (at least 6 months) of time that can cause severe social, psychological, and psychosomatic problems in the victim (8). Bullying, they reported, had also been qualified by some authors as humiliating, intimidating, or threatening; the latter two being mentioned by our respondents as what they had seen that was to be avoided. Bullying, if left unabated, has long-term economic consequences regarding recruitment and retention (8), lending credence to the respondents’ suggestion that it should be avoided. Thus, results of an earlier study suggest that involving employees across all cadres in developing and implementing participatory interventions; is likely to be successful (8). Although the cadre in this study is management cadre, this is the first step in a series of interventions which would build on the findings of this study. This is crucial as workplace violence threatens workers’ health, safety, and organizational productivity (19).

We do not find it surprising that an item of mention under the theme of administrative control included the urgent establishment of a workplace violence prevention program unit, considering some of the incidents of workplace violence some claimed to have witnessed at work; thus, commented that it was overdue in the hospital. This is a good build-up on literature that notes that an effective workplace violence prevention program/reporting system is key to preventing violence and managing the consequence of the incidents (10–12). Sanctions of erring defaulters to deter others is a strategy that appears to get things to work in our environment, so it was only fitting that our respondents strongly emphasized it as a strategy for prevention. Posting of Emergency Department (ED) signage on a safe work environment mentioned on the United States Occupational Safety and Health Administration (OSHA) list of interventions (12) (was echoed by our respondents, albeit in a modified manner- inscriptions telling people what to know/do. The understanding is that people behave better when guided or warned of the consequences of defaulting. Having a security presence and responding quickly by security personnel corroborates these strategies in the OSHA list of interventions (12). Their training and empowerment are critical as first responders to de-escalate violent incidents after the immediate witnesses. Some interventions on the OSHA list not mentioned by this study group include knowing personal de-stressors, having chaplains round with staff to see how they are doing, having alarm systems for ED workers to call for help and having a mentor to go to. Our people would not easily open up about their encounters unless they were sure their confidentiality would be maintained. The clergy in our environment command that trusts, so, surprisingly, this was not mentioned as an avenue for victims or potential victims to get counselling. Perhaps the group did not see it as an option possible in the workplace.

### Limitation

Whereas this is the first study that involved employees across all cadres who are management staff in developing strategies for preventing workplace violence in tertiary health facilities, the study has some limitations. The data may be subject to social-desirability bias, where respondents may have felt obliged to report negatively on the violence issues. However, the research assistants were well-trained to disassociate themselves from the research team and probe for all positive and negative opinions. Furthermore, although the non-use of a tape recorder (as requested by many) enabled the respondents to have their rights respected as well as allowing them to be free to make honest contributions during the discussions, some information may have been missed. However, the notetakers, being experts ensured that as much information as possible was documented. The disadvantages of purposive sampling in qualitative studies such as the inability to generalize research findings and error in judgement of the researcher in selection (researcher bias) were weighed against the fact that this was the most appropriate sampling method for this study since there is a relatively limited number of staff at management level for sampling. The possible error in judgement in the selection and elimination of important subgroups was mitigated by the objective invitation of the heads of departments (whomever they may be) as the current head of management in their departments. Also, notwithstanding the limitation of generalizability, hospitals in similar settings may find the information useful in designing their own workplace violence prevention programs.

## Conclusion

This study identified five broad intervention strategies from management-level staff to prevent workplace violence in a tertiary health facility. Respect, especially among different cadres of health workers, was the most common strategy perceived to be vital. Other strategies were discouraging intimidation of staff by staff (workplace bullying), provision of adequate and appropriate administrative controls and having adequate security presence in the hospital. As workplace violence threatens worker health and safety and organizational productivity, engaging a wider range of stakeholders and key influencers to ensure the effective development and implementation of a workplace violence prevention program for an organized response is recommended as we key into the call by ILO to prohibit and prevent violence and harassment at work.

## Data availability statement

The original contributions presented in the study are included in the article/supplementary material, further inquiries can be directed to the corresponding author.

## Ethics statement

The studies involving humans were approved by Research and Ethics Committee, Alex Ekwueme Federal University Teaching Hospital Abakaliki Nigeria. The studies were conducted in accordance with the local legislation and institutional requirements. The participants provided their written informed consent to participate in this study.

## Author contributions

AA conceptualized the study and participated in the design, supervised data collection, and drafted the manuscript. BA and AU participated in the study design, supervised data collection and analysis, and manuscript revision. BI participated in designing the study, data collection, and manuscript revision. IE participated in the literature search, data collection, data analysis and manuscript revision. FO, OO, DI-O, and UA participated in the literature search, data collection, and manuscript revision. RE participated in the design, supervised data collection, and manuscript revision. JU participated in conceptualizing and designing the study, supervised data collection, and manuscript revision. All authors contributed to the article and approved the submitted version.

## Conflict of interest

The authors declare that the research was conducted in the absence of any commercial or financial relationships that could be construed as a potential conflict of interest.

## Publisher’s note

All claims expressed in this article are solely those of the authors and do not necessarily represent those of their affiliated organizations, or those of the publisher, the editors and the reviewers. Any product that may be evaluated in this article, or claim that may be made by its manufacturer, is not guaranteed or endorsed by the publisher.
